# Succession intention and environmental investment: The moderating role of social status

**DOI:** 10.3389/fpsyg.2022.972565

**Published:** 2022-09-12

**Authors:** Qi Zhang, Lei Xiong, Youliang Yan, Zengji Song, Zezhou Wen

**Affiliations:** ^1^School of Economics and Business Administration, Chongqing University, Chongqing, China; ^2^Finance and Accounting R&D Centre, Chongqing University of Technology, Chongqing, China; ^3^School of Economics, Huazhong University of Science and Technology, Wuhan, China

**Keywords:** environmental investment, family firms, management succession intention, ownership succession intention, social status

## Abstract

Drawing on expectancy theory, this study explains how founders’ succession intentions might influence family firms’ environmental environments. Using a nationally representative sample of Chinese private firms, we find that family firms make more environmental investments when founders have succession intentions. We also find that the relationship between founders’ succession intentions and family firms’ environmental investments is negatively moderated by the founders’ subjective social status. Moreover, the results show that, compared with ownership succession intentions, the positive role of founders’ management succession intentions on family firms’ environmental investments is more prominent. This study helps us to better understand the impact of succession intentions on family business decision-making from a psychological perspective. It enriches the research on succession intentions and provides practical implications for family firms’ sustainable development.

## Introduction

Family firms are not only the most prevalent business type around the world but also a norm in Asian countries (Claessens et al., 2000). The Chinese mainland has experienced dramatic growth since the reform and opening up in 1978. After 40 years of development, in 2018, Chinese family firms accounted for 85% of the private enterprises in China^1^ and have played an essential role in job creation, technological innovation, and tax contributions. However, as the first-generation founders age, many family firms in China are now faced with the challenge of generational succession. In the United States, only about 40% of family-owned businesses have transitioned to the second generation (see footnote 1). Intergenerational succession is a top priority for family businesses to maintain family control and is a critical choice for a firm’s long-term development and continuance (Cao et al., 2015). In this study, we take private family firms as our unit of analysis to examine the effects of founders’ succession intentions on firms’ environmental protection behaviors.

Both developed and developing countries are facing the problem of environmental degradation. Green development has become an important component of global environmental governance (Maggioni and Santangelo, 2017). As the major contributor to environmental pollution (Huang and Lei, 2020; Bendell, 2021), firms’ efforts in pollution control are important to the transition towards a “green economy” (Liu et al., 2022; Tian et al., 2022b). Most environmental research focuses on listed firms and is concerned with the influence of environmental regulations and governance on a firm’s environmental investment. For example, Zeng et al. (2020) demonstrated the influence of implementing environmental responsibility audits on firms’ environmental protection investments. Tian et al. (2021) empirically tested the positive impact of cross-shareholding on corporate environmental investments. Xu and Yan (2019) illustrated the significant positive links between political connections and corporate environmental investments. However, few studies have analyzed environmental investments by private family firms from the perspective of intergenerational succession.

Intergenerational succession is a key distinctive feature of family firms. During the intergenerational inheritance stage, these companies tend to focus more on non-financial goals and pursue the long-term survival of the family (Sharma et al., 2003). As an important manifestation of environmental responsibility, environmental investment improves the social image of family firms and the legitimacy of successors (Yang et al., 2022). In this study, we use a nationally representative sample of Chinese family firms to empirically test the relationship between founders’ succession intentions and family firms’ environmental investments. We also consider how a founder’s perceived social status affects the relationship between their succession intention and the tendency to invest in pollution control. Aside from institutional pressures, environmental investments can also be driven by “preconscious acceptance of institutionalized values or practices” (DiMaggio, 1988: 17) or social expectations and norms that outline good behavior patterns (Berrone et al., 2010). Due to “being in the spotlight,” high-social-status entrepreneurs face high stakeholder expectations and are subject to intense scrutiny of their corporate social responsibility (CSR) activities (Liu et al., 2021). Some research investigates the relationship between the social status of entrepreneurs and CSR activities focused on firms’ donation engagement (Li et al., 2015; Liu et al., 2021; Niu et al., 2021). We explore the moderating role of social status in the relationship between succession intentions and environmental investments.

This study makes several contributions to the literature. First, drawing on expectancy theory, which is one of the most commonly used theories of motivation in the field of organizational psychology, this study helps us to better understand the impact of succession intentions on family business decision-making from the perspective of social responsibility in environmental investment. It supports expectancy theory with empirical evidence and enriches the literature on the economic consequences of succession intentions. Second, our findings add to the environmental literature because most environmental studies that explore the factors influencing a firm’s environmental strategies focus on institutional pressures and benefits from compliance with regulations. In contrast, we examine the relationship between founders’ succession intentions and family firms’ environmental investments, which expands the scope of research on the factors influencing environmental investments. Third, we noticed the moderating role of the founder’s perceived social status in the relationship between the founder’s succession intentions and the family firm’s environmental investments. The social status of Chinese business founders has greatly improved in recent decades, which provides a unique context for understanding how social status influences entrepreneurs’ strategic decisions on environmental investments.

The remainder of this paper is organized as follows. Section “Literature review and hypothesis development” reviews related literature and presents our hypotheses. Section “Data and empirical methods” describes the data, defines the variables, and presents descriptive statistics and models. Section “Empirical results” reports the empirical results and the results of the robustness tests. Section “Conclusion” concludes the paper.

## Literature review and hypothesis development

### Succession intention

Scholars proposed the socioemotional wealth (SEW) model to analyze the corporate behavior of family firms. The SEW model suggests that family firms’ strategic decisions are made to preserve their SEW (Berrone et al., 2012). SEW refers to non-economic utilities, such as “family control and influence, identification of family members with the firm, binding social ties, emotional attachment of family members, and renewal of family bonds to the firm through dynastic succession” (Berrone et al., 2012: 259). In pursuit of these affective endowments, family firms display a stronger preference for non-economic but socially worthy activities than do non-family firms (Berrone et al., 2010). Several empirical studies have confirmed these findings. For example, Li et al. (2015) suggest that family-controlled firms engage in philanthropic activities to maintain SEW. Gómez-Mejía et al. (2007) show that preference for family control takes priority over higher returns. Schulze et al. (2003) report altruistic conduct for family members in family firms. Intergenerational succession, as a critical dimension of SEW, is of great significance to family firms. There are only a small proportion of family firms that could survive the transition from the first to the second generation (Shen and Su, 2017).

Much of the empirical analysis on intergenerational succession has been devoted to understanding the impacts of family successions on firm performances and business decisions. For example, researchers have shown the vital role of family successions in R&D (Chrisman and Patel, 2012), corporate philanthropy (Li et al., 2015), and internationalization (Yang et al., 2020). However, few studies have discussed the economic consequences of succession intentions. Succession intentions focus on the willingness of founders to pass on the firms’ ownership or management to the next generation; it reflects the founders’ inclination to continue the family businesses (He et al., 2014). Several internal and external factors can influence founders’ succession intentions, such as external system environment (He et al., 2014), population policy (Cao et al., 2015) and founders’ religiosity (Shen and Su, 2017). Since founders’ succession intentions reflect their perception of firms’ long-term development, which can be predictors in inferring firms’ strategic decisions.

### Environmental investment

Environmental investment is a special type of corporate investment, including pollution-control costs, environmental improvement expenditures, and other expenses related to environmental practices (Ehresman and Okereke, 2015). In the short term, it has high costs and generates low returns (Wei and Zhou, 2020). Thus, firms often lack the incentive to make voluntary environmental investments (Tian et al., 2021). Governments around the world have introduced numerous environmental regulations and policies (Du et al., 2020) and provided various green subsidies for firms to make more environmental investments (Huang et al., 2020). Although, it is sometimes difficult to distinguish whether companies substantially address environmental issues through actions, or simply adopt green-washing strategies by engaging in symbolic communication on environmental problems (Walker and Wan, 2012). In general, environmental investment is considered “seemingly good” in the eyes of the public (Liu et al., 2021). As environmental problems worsen, firms can benefit from making pro-environmental investments. On the one hand, higher environmental investment means lower environmental compliance costs (Maxwell and Decker, 2006). On the other hand, environmental investment, as an act of social responsibility, helps to establish legitimacy, improve corporate image, stabilize partners, and attract consumers (Yang et al., 2022).

### Social status

Social status is a concept originating from sociology research and is used widely in management studies (Liu et al., 2021). An entrepreneur’s social status is defined as their standing within the social order; it is determined by factors such as education, wealth, occupation, and political power (Chen and Williams, 2018; Liu et al., 2021). The social status of Chinese business founders in the private sector had not improved until China’s economic reforms, announced in 1978 (Au and Sun, 1998). Before that, private firms were not allowed to operate in China, and private business founders were thought to be selfish (Liu et al., 2021). With loosened ideological restrictions and abandoned ownership discrimination in the 1990s, some business founders entered into the political establishment, and some government employees started their own businesses (Liu et al., 2021). This led to an improvement in the social status of Chinese entrepreneurs. With the rapid growth of family businesses after China’s economic reforms, more founders have enjoyed a relatively high social status. High-social-status entrepreneurs with respected and honored standing in a social hierarchy enjoy better access to information and resources; correspondingly, they face high expectations from stakeholders and are the targets of stringent scrutiny regarding their CSR activities (Liu et al., 2021). This experience of “being in the spotlight” is also an important part of Chinese culture. Confucianism, which has pervasive influence in China, advocates “relieving the distress of the world once one achieves eminence.” In such a unique culture, when entrepreneurs develop a high self-evaluation of social status through interaction and comparison with others in society, their perceived pressure from the public to behave in a prosocial manner increases, and they are motivated to proactively address the environmental demands of the society.

### Hypothesis development

We draw upon expectancy theory to explain how founders’ succession intentions influence family firms’ environmental investments. Expectancy theory is one of the most commonly used theories of motivation among organizational and industrial psychologists to explain the decision-making process of individuals (Vroom, 1964; Chiang and Jang, 2008; Li et al., 2015). According to expectancy theory, expectancy, instrumentality and valence are prerequisites for individuals to make decisions on various behavioral options (Fudge and Schlacter, 1999; Chiang and Jang, 2008). Expectancy measures the perceived correlation between effort and performance (Fudge and Schlacter, 1999). Instrumentality is the belief that a person’s rewards are closely tied to the level of performance (Fudge and Schlacter, 1999; Chiang and Jang, 2008). Valence refers to the subjective value placed on rewards (Chiang and Jang, 2008; Li et al., 2015). These elements combine multiplicatively to determine the motivational force for a behavior (Fudge and Schlacter, 1999; Chiang and Jang, 2008; Li et al., 2015).

When founders are willing to pass on their business to the next generation, the family firm is not only an asset that can be easily sold but also a symbol of the family’s heritage and traditions (Chen and Chen, 2014). Founders expect to maintain the values and vision of the family through family firms (Chen and Chen, 2014), and they are inclined to take action to preserve the positive image of the firm for future generations. Thus, founders with succession intentions tend to have a long-term vision and place great value on SEW (Li et al., 2015). As environmental issues become increasingly important, environmental investment, as an act of social responsibility, has been documented to improve corporate social reputation (Aksak et al., 2016), establish legitimacy (Yang et al., 2022), and gain the trust of stakeholders (Tian et al., 2022a), which contributes to corporate sustainable development (Tian et al., 2020). Based on these arguments, it can be inferred that family firms are able to benefit from environmental investments, and the benefits are conducive to family firms’ long-term continuance, which is of great significance to founders with succession intentions. In the language of expectancy theory, the motivation force for making environmental investments is high when founders are willing to pass on their business to the next generation; because the expectancy, instrumentality and valence, which are the prerequisites underlying founders’ motivation to engage in environmental investments are high. Therefore, founders with succession intentions are motivated to make environmental investments.

Moreover, environmental investment usually takes a long time to materialize (Russo and Harrison, 2005). The long-term vision attached to family firms with succession intentions helps generate patient capital, which is required by environmentally friendly policies. Hence, our first hypothesis is as follows:

*H1:* Family firms make more environmental investments when founders have succession intentions than when they do not.

Entrepreneurs’ social status has been documented to affect firms’ strategic decisions (Chen et al., 2012; Liu et al., 2021). We argue a weaker positive relationship between the founder’s succession intention and the family firm’s environmental investments among founders with a higher subjective social status, compared to those with a lower subjective social status for two reasons. First, society assigns appropriate norms of behavior to different social classes, and in many cases, adherence to social norms of behavior becomes a prerequisite of positive social image and reputation (Niu et al., 2021). Meanwhile, high-social-status entrepreneurs are judged stringently by stakeholders (Merton, 1968), and they are easily recognized and criticized in terms of social irresponsibility (Liu et al., 2021). As a result, founders with a higher perceived social status are faced with greater pressure exerted by these social norms, and are more likely to make environmental investments. Second, founders with higher subjective social status tend to show higher levels of psychological security, and they tend to be more optimistic, confident, and have better self-control (Niu et al., 2021). They may perceive their firms’ ability to engage in CSR activities to be higher than that of their peers of lower social status. Thus, they are less likely to be irresponsible regarding environmental issues. In sum, a higher subjective social status might serve as the intrinsic motivations of entrepreneurs to make more environmental investments. Based on this, we propose the following hypothesis:

*H2:* Compared to founders with a lower subjective social status, among those with a higher subjective social status, there is a weaker positive relationship between the founder’s succession intention and the family firm’s environmental investments.

According to Bennedsen et al. (2015:7), there are three modes of ownership-control transition for family firms: “family succession of both ownership and management, family ownership with professional management, and exit.” Due to the poorly developed financial markets and the under-development of the external managerial markets, Chinese family firms are faced with a higher threshold of management professionalization (Cao et al., 2015). Meanwhile, the non-financial aspects of the firm that satisfy the family’s emotional needs (Gómez-Mejía et al., 2007), such as family reputation (Zellweger et al., 2013), social ties (Berrone et al., 2012), and good relationships with stakeholders (Bennedsen et al., 2015), are not easily transferred to outside professional managers. Thus, in most cases, the family succession of ownership and management takes precedence over the family succession of ownership with firms managed by outsiders (Cao et al., 2015). If founders intend to transfer both control and ownership to younger heirs, we refer to this as management succession intention. On the other hand, ownership succession intention refers to the founder’s expectation that the next generation will succeed to business ownership but will not manage their own firm (Shen and Su, 2017).

Within a firm, all actors are guided by self-interest (Berrone et al., 2010). Their divergent goals lead to contested objectives, and the ultimate decision depends on the interests of the controlling party (Berrone et al., 2010). For most Chinese family firms, substantial discretion is enjoyed by the founder’s family because ownership and management are not separate; family management and decision procedures usually take the place of business decision procedures (Cao et al., 2015). The controlling power of the family elevates its right to pursue SEW through substantive responses to environmental demands, which may not be of interest to professional managers who are responsible for profit maximization (Zeng et al., 2020). Thus, founders’ expectations of having the next generation succeed to both ownership and management indicate that a firm’s environment-friendly policies are more likely to be consistent over a longer period of time. With both ownership and control handed over to younger heirs, firms’ environmental strategies are less likely to face conflicts of interest between successors and external managers in the future. The anticipation of uninterrupted environmental commitment after a dynastic transition is in favor of the founder’s current environmental investments. Accordingly, we differentiate between management succession and ownership succession, and propose Hypothesis 3:

*H3:* The positive relationship between a founder’s succession intention and a family firm’s environmental investment is more prominent when founders have management succession intentions than ownership succession intentions.

## Data and empirical methods

### Data

We obtained data from the 12th Chinese Private Enterprises Survey^2^ (CPES) conducted in 2016. The CPES is conducted by a research team, whose member organizations include the United Front Work Department of the Chinese Communist Party Central Committee, the All-China Federation of Industry and Commerce, the State Administration for Market Regulation, the Chinese Academy of Social Sciences, and the Chinese Private Economy Research Association. As a representative large-scale social survey, the CPES collects individual-and firm-level information (Chen et al., 2019). It aims to understand the thoughts, opinions, and requirements of private firm founders and to identify the problems confronting private firms (Jiang et al., 2015). The history and survey method of CPES are comprehensively demonstrated by Chen et al. (2019), so we do not elaborate on them here. In the 12th CPES, there were 8,111 cases. The definition of family firms may differ in the literature; in line with Li et al. (2015), we define family firms as firms with at least 50% family ownership. We use regression analyses to test our hypotheses. We describe all of our variables in more detail in the next section.

### Variables

#### Dependent variable

Our dependent variable (*Env*) is a firm’s environmental investments. Referring to Xu and Yan (2019), we measure a firm’s environmental investments as their pollution-control investments in 2015, scaled by sales.

#### Independent variables

Following Li et al. (2015) and Shen and Su (2017), our independent variables are succession intention (*SI*), management succession intention (*SI_M*), and ownership succession intention (*SI_O*). In the survey, founders were asked whether they intended to allocate shares to their children or to have them manage the family firm. *SI* is a dummy variable that equals one if the founder expects to pass ownership or management control to the next generation. *SI_M* and *SI_O* are two dummy variables; *SI_M* equals one if the founder has management succession intentions, and *SI_O* equals one if the founder has ownership succession intentions.

#### Moderating variable

We take the founder’s subjective social status (*SS*) as our moderating variable. According to Weber (1947), a social actor’s social status consists of economic, social, and political status, and these elements can be measured by one’s wealth, reputation, and power, respectively. In the survey, founders were required to estimate their economic, social, and political hierarchy on a 10-point scale (1 indicates the highest position, 10 indicates the lowest position). Referring to Liu et al. (2021) and Niu et al. (2021), we reversed the values, and *SS* was measured as the mean of the three values.

#### Control variables

We controlled for the founder’s gender, age, educational background, and political connections. We also controlled for the shareholding of the founder and their family as a proxy for family ownership. Firm size and profitability were also included in the model, as were industry dummies; firms in the financial industry were excluded. The measurements for these variables are listed in Table 1.

**Table 1:** Definition and measurement of variables.

Variables	Measurement
*Env*	The ratio of pollution-control investments to sales
*SI*	Value of 1 if the founder intends to give ownership or management control to the next generation; otherwise, 0
*SS*	Mean of the economic, social, and political 10-point scale measures, reverse-coded
*SI_M*	Value of 1 if the founder has management succession intention; otherwise, 0
*SI_O*	Value of 1 if the founder has ownership succession intention; otherwise, 0
*Gender*	Value of 1 if the founder is a male; otherwise, 0
*Age*	The natural logarithm of the founder’s age
*Education background*	Value of 1 assigned to junior school or below, 2 to senior high school, 3 to junior college, 4 to bachelor’s degree, 5 to master’s degree, 6 to doctorate
*Political connection*	Value of 1 if the founder is a member of the National People’s Congress or the Chinese People’s Political Consultative Conference; otherwise, 0
*Ownership*	Proportion of shares held by the controlling family
*Firm size*	The natural logarithm of sales
*Profitability*	The ratio of net profits to sales

### Descriptive statistics and correlation analysis

The descriptive statistics of the founder-and firm-specific variables are shown in Table 2. Cases with missing data were deleted, and all continuous variables were winsorized at the 1% and 99% levels. Table 2 shows that, in general, our sample of private family firms has a low level of environmental investment. The average value of pollution-control costs to sales is only 0.6%. However, the standard deviation of *Env* is more than three times larger than its mean, indicating that environmental investment among the family firms in our sample varies widely. Furthermore, 16.7% of the founders in our sample have succession intentions; the proportion of founders with management succession intentions is higher than that of founders with ownership succession intentions. On average, the value of the founder’s subjective social status is 5.066, and the standard deviation (1.785) is low. In terms of other descriptive characteristics, 81.7% of the family founders are male, and their average age was 46 years. At least half of the founders in our sample attended junior college or higher; 30.5% of our founders had political connections, and at least half of our firms were wholly owned by the founder’s family.

**Table 2:** Descriptive statistics.

Variables	Observations	Mean	Median	Std. Dev.	Min	Max
*Env*	3,382	0.006	0.000	0.022	0.000	0.167
*SI*	3,382	0.167	0.000	0.372	0.000	1.000
*SS*	3,382	5.066	5.000	1.785	1.000	10.000
*SI_M*	3,382	0.151	0.000	0.358	0.000	1.000
*SI_O*	3,382	0.015	0.000	0.123	0.000	1.000
*Gender*	3,382	0.817	1.000	0.387	0.000	1.000
*Age*	3,382	3.821	3.850	0.208	2.996	4.394
*Education background*	3,382	2.750	3.000	1.091	1.000	6.000
*Political connection*	3,382	0.305	0.000	0.461	0.000	1.000
*Ownership*	3,382	0.916	1.000	0.156	0.500	1.000
*Firm size*	3,382	6.078	6.176	2.577	0.788	12.087
*Profitability*	3,382	0.122	0.058	0.305	−1.500	1.000

In addition, Table 3 reports the pairwise correlation coefficients of all variables. *SI* and *SI_M* are highly correlated, but they are not used in the same model. Although, many of the correlations are statistically significant, the magnitudes of these correlations are substantively small, indicating that our model does not suffer from serious multicollinearity problems.

**Table 3:** Correlations.

Variables	(1)	(2)	(3)	(4)	(5)	(6)	(7)	(8)	(9)	(10)	(11)	(12)
*(1) Env*	1.000											
*(2) SI*	0.049^***^	1.000										
*(3) SS*	0.018	0.157^***^	1.000									
*(4) SI_M*	0.041^**^	0.944^***^	0.150^***^	1.000								
*(5) SI_O*	0.028^*^	0.280^***^	0.038^**^	−0.053^***^	1.000							
*(6) Gender*	0.009	0.019	0.079^***^	0.022	−0.009	1.000						
*(7) Age*	0.043^**^	0.305^***^	0.230^***^	0.305^***^	0.038^**^	0.062^***^	1.000					
*(8) Education background*	−0.028^*^	−0.074^***^	0.185^***^	−0.087^***^	0.029^*^	0.031^*^	−0.142^***^	1.000				
*(9) Political connection*	0.039^**^	0.151^***^	0.388^***^	0.144^***^	0.037^**^	0.085^***^	0.281^***^	0.219^***^	1.000			
*(10) Ownership*	−0.038^**^	0.023	−0.049^***^	0.018	0.016	−0.043^**^	−0.035^**^	−0.085^***^	−0.048^***^	1.000		
*(11) Firm size*	−0.030^*^	0.207^***^	0.434^***^	0.202^***^	0.040^***^	0.158^***^	0.280^***^	0.334^***^	0.493^***^	−0.126^***^	1.000	
*(12) Profitability*	0.016	−0.055^***^	−0.069^***^	−0.055^***^	−0.006	−0.044^**^	−0.082^***^	−0.125^***^	−0.100^***^	0.018	−0.223^***^	1.000

*** *p* < 0.01;

** *p* < 0.05;

* *p* < 0.10.

### Regression method

To test the effect of founders’ succession intentions on family firms’ environmental investments and the moderating effect of social status, we estimate the following regression models:


(3.1) $$Env=\beta_{0}+\beta_{1}*SI+\beta_{2}*SS+\sum \gamma_{i}*ControlVariables+\varepsilon$$

(3.2) $$\begin{array}{c} Env=\beta_{0}+\beta_{1}*SI+\beta_{2}*SS+\beta_{3}*\left(SI*SS\right)\\ +\sum \gamma_{i}*ControlVariables+\varepsilon \end{array}$$

In our sample, data on family firms’ environmental investments are characterized by many zeros, so we fitted a tobit model to run the regression analyses.

## Empirical results

### Regression results for founders’ succession intentions and family firms’ environmental investments

Table 4 presents the regression results. Model 1 uses *Env* as the dependent variable. The main independent variable of interest is the founder’s succession intention. We also include other founder-and firm-specific variables as control variables along with the industry dummies. Consistent with H1, Model 1 of Table 4 shows that family firms with succession intentions make more environmental investments than family firms without succession intentions. We also find that family firms have a higher ratio of pollution-control investments to sales when founders have a higher subjective social status.

**Table 4:** Empirical results.

Model	(1)	(2)	(3)	(4)	(5)	(6)
Dependent variable	*Env*	*Lnenv*	*Dummyenv*	*Env*	*Env*	*Env*
Subgroup					*SS* ≥ mean *SS*	*SS* < mean *SS*
*SI*	0.006^**^	0.306^*^	0.127^*^	0.032^***^	0.003	0.015^**^
	(0.003)	(0.163)	(0.069)	(0.009)	(0.003)	(0.006)
*SS*	0.001^*^	0.089^**^	0.037^**^	0.002^***^		
	(0.001)	(0.041)	(0.017)	(0.001)		
SI*SS				−0.005^***^		
				(0.002)		
*Gender*	0.003	0.292	0.102	0.003	0.003	0.004
	(0.003)	(0.179)	(0.071)	(0.003)	(0.004)	(0.006)
*Age*	0.007	0.356	0.102	0.006	0.011	−0.001
	(0.006)	(0.363)	(0.146)	(0.006)	(0.008)	(0.011)
*Education background*	−0.001	−0.085	−0.043	−0.001	−0.001	−0.001
	(0.001)	(0.064)	(0.026)	(0.001)	(0.001)	(0.002)
*Political connection*	0.009^***^	0.648^***^	0.244^***^	0.010^***^	0.007^***^	0.014^**^
	(0.003)	(0.147)	(0.061)	(0.003)	(0.003)	(0.006)
*Ownership*	−0.019^***^	−0.922^**^	−0.364^**^	−0.019^***^	−0.010	−0.036^***^
	(0.007)	(0.387)	(0.162)	(0.007)	(0.008)	(0.013)
*Firm size*	0.003^***^	0.625^***^	0.184^***^	0.003^***^	0.002^***^	0.005^***^
	(0.001)	(0.036)	(0.014)	(0.001)	(0.001)	(0.001)
*Profitability*	0.002	0.386	0.060	0.002	0.004	−0.001
	(0.004)	(0.260)	(0.112)	(0.004)	(0.005)	(0.007)
*Industry*	Yes	Yes	Yes	Yes	Yes	Yes
*Constant*	−0.075^***^	−6.813^***^	−2.138^***^	−0.077^***^	−0.072^**^	−0.057
	(0.025)	(1.482)	(0.600)	(0.025)	(0.031)	(0.045)
*N*	3,382	3,382	3,382	3,382	1,678	1,704
*Pseudo R^2^*	−0.356	0.152	0.225	−0.363	−0.126	−21.672

Standard errors are in parentheses.

*** *p* < 0.01;

** *p* < 0.05;

* *p* < 0.10.

We verify the robustness of these results using alternative measures of the dependent variable in Models 2 and 3. Model 2 of Table 4 measures the family firm’s environmental investments using *Lnenv*, which is the natural log of one plus the pollution-control investments a firm made in 2015. The coefficient on *SI* is positive and statistically significant, which means that the absolute amount of family firms’ environmental investments is larger when founders have succession intentions. This result is consistent with that reported in Model 1. Model 3 uses *Dummyenv* as the dependent variable; it is equal to one if a firm made an environmental investment in 2015 and zero otherwise. We fitted a probit model to run the regression. The coefficient on *SI* is still positive and statistically significant, which means that family firms are more likely to make environmental investments when founders have succession intentions. These results support H1.

### Regression results for the moderating effect of founders’ subjective social status

**Figure 1:**
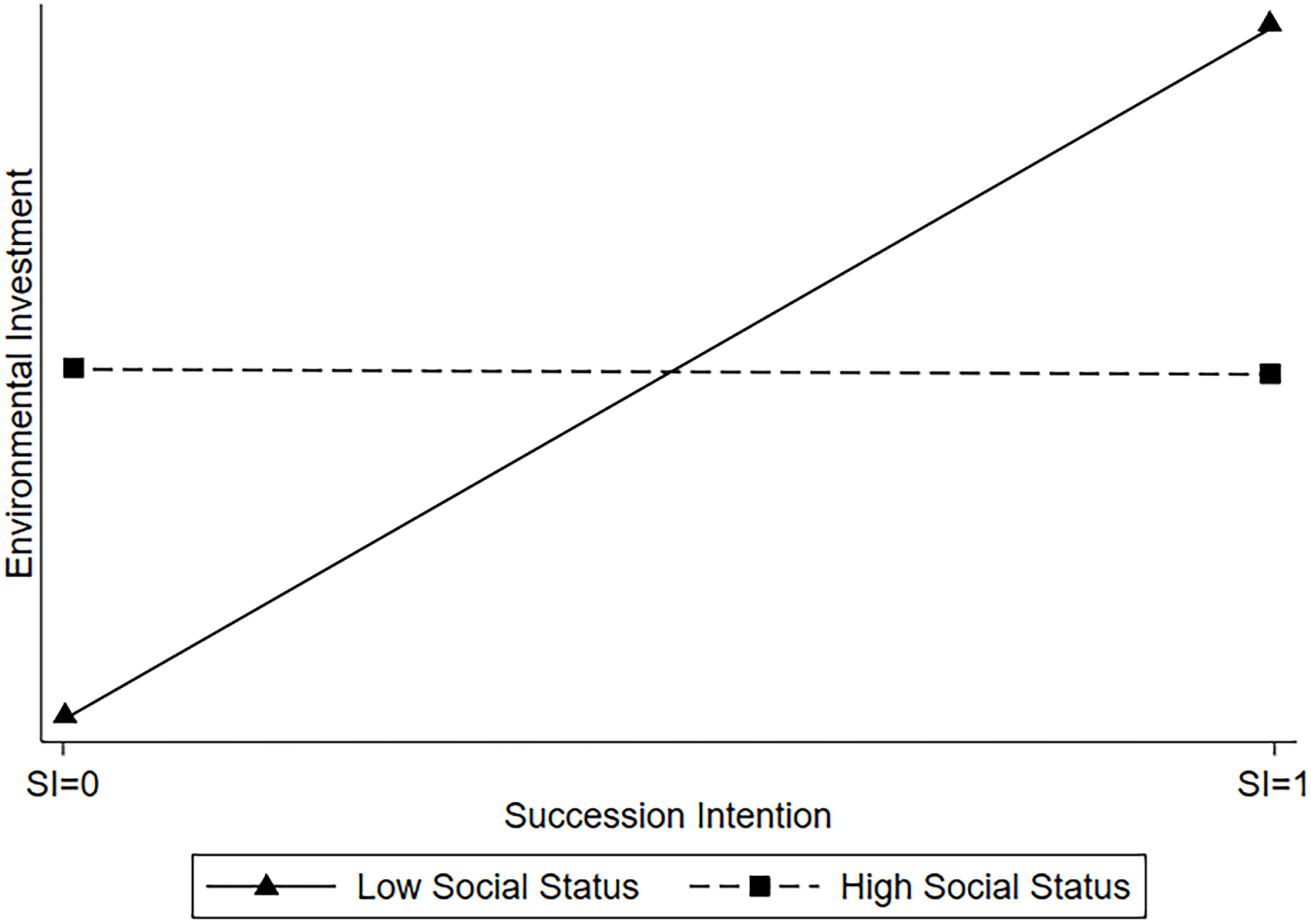
The moderating effects of founders’ subjective social status.

Model 4 is similar to Model 1 but includes the interaction term between the founder’s succession intention and subjective social status. The interaction term is negative and significant (*β* = −0.005, *p* < 0.01), which is consistent with H2. For founders with a higher subjective social status, there is a weaker positive relationship between the founder’s succession intention and the family firm’s environmental investments. The moderating effects are shown in Figure 1. The slope of the line for founders with high subjective social status (i.e., a status that is one standard deviation above the mean) is flatter than that of those with low subjective social status (i.e., a status that is one standard deviation below the mean). For founders with a lower subjective social status, the impact of the founder’s succession intention on a firm’s environmental investment is more prominent. Therefore, a founder’s subjective social status negatively moderates the positive relationship between their succession intention and a firm’s environmental investments.

We test the robustness of the moderating effects of the founder’s subjective social status on the relationship between the founder’s succession intention and the family firm’s environmental investments using seemingly unrelated regression. We divide our sample into two subgroups according to the level of the founder’s self-evaluated social status. Model 5 in Table 4 reports the regression results for family firms whose founders have a subjective status no less than the mean of *SS*. In comparison, Model 6 in Table 4 shows the regression results for family firms whose founders have a subjective social status less than the mean of *SS*. We tested whether the coefficients on *SI* in these two subgroups were equal. The *p* value is 0.085; it is estimated based on the null hypothesis that the coefficients on *SI* in Models 5 and 6 are equal. This suggests that the coefficient estimate for *SI* in Model 6 is significantly different from that in Model 5. In other words, when founders have a lower subjective social status, the positive effects of their succession intention on their firm’s environmental investments are more prominent. These results are consistent with those reported in Model 4 and support H2.

### Comparison between management succession intention and ownership succession intention

We differentiate between management succession and ownership succession, and identify the different effects of the founder’s intentions for each on their firm’s environmental policies. Model 7 in Table 5 shows the regression results. Model 7 is similar to Model 1 but uses two succession intention dummy variables to replace the variable *SI*: *SI_M* and *SI_O*. The parameter coefficient of *SI_M* is positive and statistically significant, while the positive parameter coefficient of *SI_O* is not statistically significant. This finding suggests that, compared with ownership succession intention, the positive relationship between founders’ management succession intention and family firms’ environmental investment is more prominent. It supports H3.

**Table 5:** Comparison between management succession intention and ownership succession intention.

Model	(7)	(8)
Dependent variable	*Env*	*Env*
*SI_M*	0.006^**^	0.036^***^
	(0.003)	(0.010)
*SI_O*	0.010	−0.018
	(0.008)	(0.033)
*SS*	0.001^*^	0.002^***^
	(0.001)	(0.001)
SI_M*SS		−0.005^***^
		(0.002)
SI_O*SS		0.005
		(0.005)
*Gender*	0.003	0.003
	(0.003)	(0.003)
*Age*	0.007	0.007
	(0.006)	(0.006)
*Education background*	−0.001	−0.001
	(0.001)	(0.001)
*Political connection*	0.009^***^	0.009^***^
	(0.003)	(0.003)
*Ownership*	−0.019^***^	−0.019^***^
	(0.007)	(0.007)
*Firm size*	0.003^***^	0.003^***^
	(0.001)	(0.001)
*Profitability*	0.002	0.002
	(0.004)	(0.004)
*Industry*	Yes	Yes
*Constant*	−0.075^***^	−0.078^***^
	(0.025)	(0.025)
*N*	3,382	3,382
*Pseudo R^2^*	−0.356	−0.366

Standard errors are in parentheses.

*** *p* < 0.01;

** *p* < 0.05;

* *p* < 0.10.

H3 was further verified using propensity score matching analysis. We fit a logistic model to predict each subject’s propensity score, using the covariates gender, age, educational background, political connection, ownership, firm size, and profitability. To better predict propensity scores, we included both linear and quadratic terms in the estimation function. The average treatment effects are presented in Table 6. Management succession intention has a positive and significant effect on a family firm’s environmental investment, which is assessed by the proportion of pollution-control investments to sales in 2015. However, the results for ownership succession intention are not significant. This suggests that the positive relationship between a founder’s succession intention and a family firm’s environmental investment is more prominent when founders have management succession intentions than ownership succession intentions. The findings support H3.

**Table 6:** The average treatment effect using propensity score matching.

	ATE	ATE
	*SI_M* (1 vs. 0)	*SI_O* (1 vs. 0)
*Env*	0.004^*^	0.004
	(0.002)	(0.004)

Standard errors are in parentheses. ATE, Average treatment effect.

* *p* < 0.10.

Model 8 of Table 5 is similar to Model 7 but includes the interaction term between the founder’s management succession intention and their subjective social status, and the interaction term between the founder’s ownership succession intention and their subjective social status. The parameter coefficient of the interaction term between management succession intention and social status is negative and statistically significant. However, neither the parameter coefficient of ownership succession intention nor the interaction term between ownership succession intention and subjective social status is statistically significant. Specifically, the negative moderating effect of a founder’s subjective social status only applies to the positive relationship between the founder’s management succession intention and the firm’s environmental investments.

## Conclusion

Using the lens of psychology, we explain how founders’ succession intentions influence family firms’ environmental investments. The main finding of this study is that Chinese family firms whose founders have succession intentions make more environmental investments than firms whose founders do not have succession intentions. It supports expectancy theory with empirical evidence. Moreover, it is observed that the founder’s subjective social status negatively moderates the positive relationship between succession intention and environmental investments. Specifically, the positive relationship between succession intention and environmental investments is weaker for founders with high subjective social status than for those with low subjective social status. When differentiating between a founder’s management and ownership succession intentions, we find that the positive effects of succession intention on a firm’s environmental investments are driven mainly by the founder’s management succession intentions. Moreover, the negative moderating effects of the founder’s subjective social status only apply to the relationship between the founder’s management succession intention and the family firm’s environmental investments. Our study enriches the extant environmental research as well as family business studies.

Our findings have several important implications. First, this study expands the scope of research on the factors influencing environmental investments. Except for legal regulations, family firms’ dynastic transition plans can affect their environmental strategies. Government departments can help to establish an institutional environment that improves founders’ willingness to pass on family businesses to the next generation to promote sustainable development of family firms. The long-term vision of family firms is conducive to firms’ environmental investments. Second, high-social-status entrepreneurs face high expectations from stakeholders, they adhere to appropriate social norms of behavior to obtain a positive social image. For founders without succession intentions, a credible public ranking list can help them identify their position in the social hierarchy and guide their self-evaluation of social status, which, in turn, could also encourage their responses to environmental issues. Thus, government departments can use such tools and encourage public scrutiny of corporate pollution practices to drive corporate environmental investments.

This study has some limitations. First, we do not use panel data, and the cross-sectional design may weaken the robustness of our findings. Second, we focus only on the moderating role of the founder’s subjective social status; other institutional-, industry-, firm-, and individual-level variables may also affect the relationship between the founder’s succession intention and the firm’s environmental investments. These limitations provide opportunities for future research in this area.

## Data availability statement

The datasets analyzed for this study are available on the website: https://cpes.zkey.cc/index.jsp. Further inquiries can be directed to the corresponding author.

## Author contributions

QZ, LX, YY, ZS, and ZW contributed to conception and design of the study. LX organized the database. QZ performed the statistical analysis. All authors contributed to the article and approved the submitted version.

## Funding

This research was supported by the National Social Science Foundation of China (grant number: 20CJY040).

## Conflict of interest

The authors declare that the research was conducted in the absence of any commercial or financial relationships that could be construed as a potential conflict of interest.

## Publisher’s note

All claims expressed in this article are solely those of the authors and do not necessarily represent those of their affiliated organizations, or those of the publisher, the editors and the reviewers. Any product that may be evaluated in this article, or claim that may be made by its manufacturer, is not guaranteed or endorsed by the publisher.

## Footnotes

^1^Relevant data come from the Global Family Business Research Centre; for details, please refer to http://eng.pbcsf.tsinghua.edu.cn/portal/list/index/id/123.html.

^2^Research Centre for Private Enterprises at Chinese Academy of Social Sciences (RCPE-CASS) is the authorized organization that manages and issues the survey data. We appreciate the data support from the above organizations. Any political issue caused by contexts is the sole responsibility of the authors.
